# Supplementary material for: Experimental introgression in Drosophila: Asymmetric postzygotic isolation associated with chromosomal inversions and an incompatibility locus on the X chromosome
Source: Mol Ecol. 2022 Dec 22;32(4):854–66. doi: 10.1111/mec.16803 (PMC10107139; doi:10.1111/mec.16803)
Supplement: Supplementary file 1 — Appendix S1 [file MEC-32-854-s001.docx]

**Supporting information**

**Experimental introgression in *Drosophila*: asymmetric postzygotic isolation associated with chromosomal inversions and an incompatibility locus on the X chromosome**

Supporting Tables ………………………………………………………………………. 1

Supporting Figures ……………………………………………………………………… 5

Supporting References ……………………………………………………………….. 9

**Supporting Tables:**

Table S1. Number and mean length of Illumina paired-end reads of each pool before and after filtering, as well as read depth (coverage) after mapping to *D. montana* and *D. flavomontana* reference genomes.

Table S2. Genomic coordinates of chromosomal inversions fixed between *D. montana* and *D. flavomontana* (Poikela et al., in prep.). Presence of inversions was checked from parental female pools, and they are illustrated against *D. flavomontana* and *D. montana* reference genomes.

Table S3. Fertile and viable F_1_ and BC_1_ females, produced by a single-pair cross of *D. flavomontana* female and *D. montana* male, contributed to the production of the sequenced BC_2_ females. These numbers were used to repeat the experiment *in silico* (simulations).

Table S4. Summary of the effect of chromosome on the number of differentially fixed SNPs between *D. montana* and *D. flavomontana*. Significant P-values are in bold. The data were analysed twice, using both *D. flavomontana* and *D. montana* reference genomes.

Table S5. Summary of the effect of genomic region (collinear, inverted) on the number of differentially fixed SNPs between *D. montana* and *D. flavomontana*. Significant P-values are in bold. The data were analysed twice, using both *D. flavomontana* and *D. montana* reference genomes.

Table S6. Information on the chromosome length (bp), the number and proportion (%) of differentially fixed SNPs (diagnostic SNPs) between parental species (*D. montana* and *D. flavomontana*) for different genomic regions, the observed mean hybrid index (HI) for different genomic regions and for both 2^nd^ generation backcross pools (BC2mon and BC2fla), and the number and proportion (%) of SNPs that showed no introgression between species (HI=0). The data were analysed twice, using both *D. flavomontana* and *D. montana* reference genomes.

**Supporting Figures:**


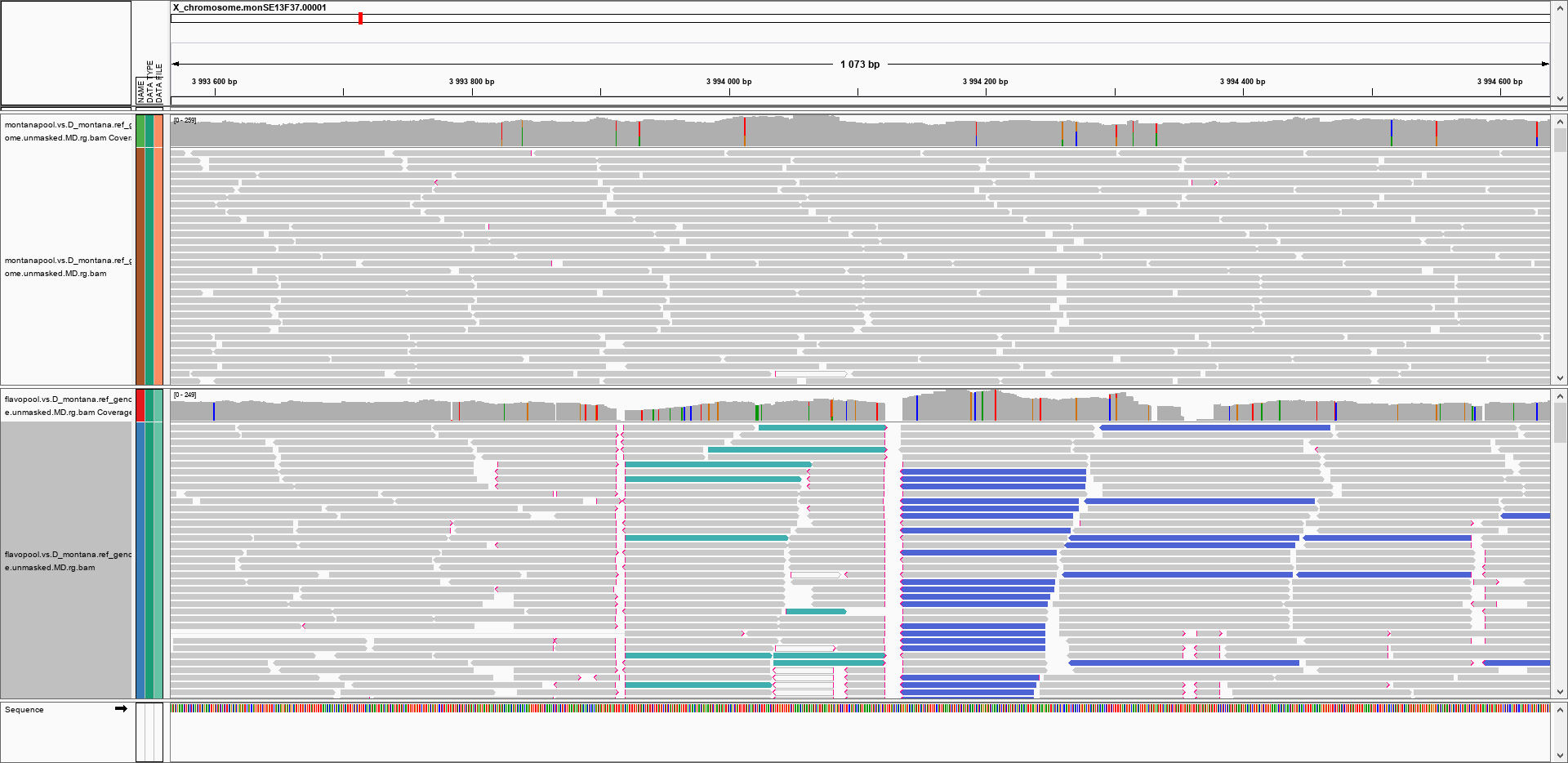


Figure S1. Example plot of an inversion breakpoint between *D. montana* and *D. flavomontana* illustrated with Interactive Genomics Viewer (Thorvaldsdóttir et al. 2012). Illumina reads of the parental strains (monSE13F37 above and flaMT13F11 below) are mapped against *D. montana* chromosome level reference genome. *D. montana* reads map well on the *D. montana* genome, while *D. flavomontana* reads split or are in reversed orientation. Red tip of a read represents the split of the read, and blue and green reads represent reads that are in reversed orientation relative to the reference genome.


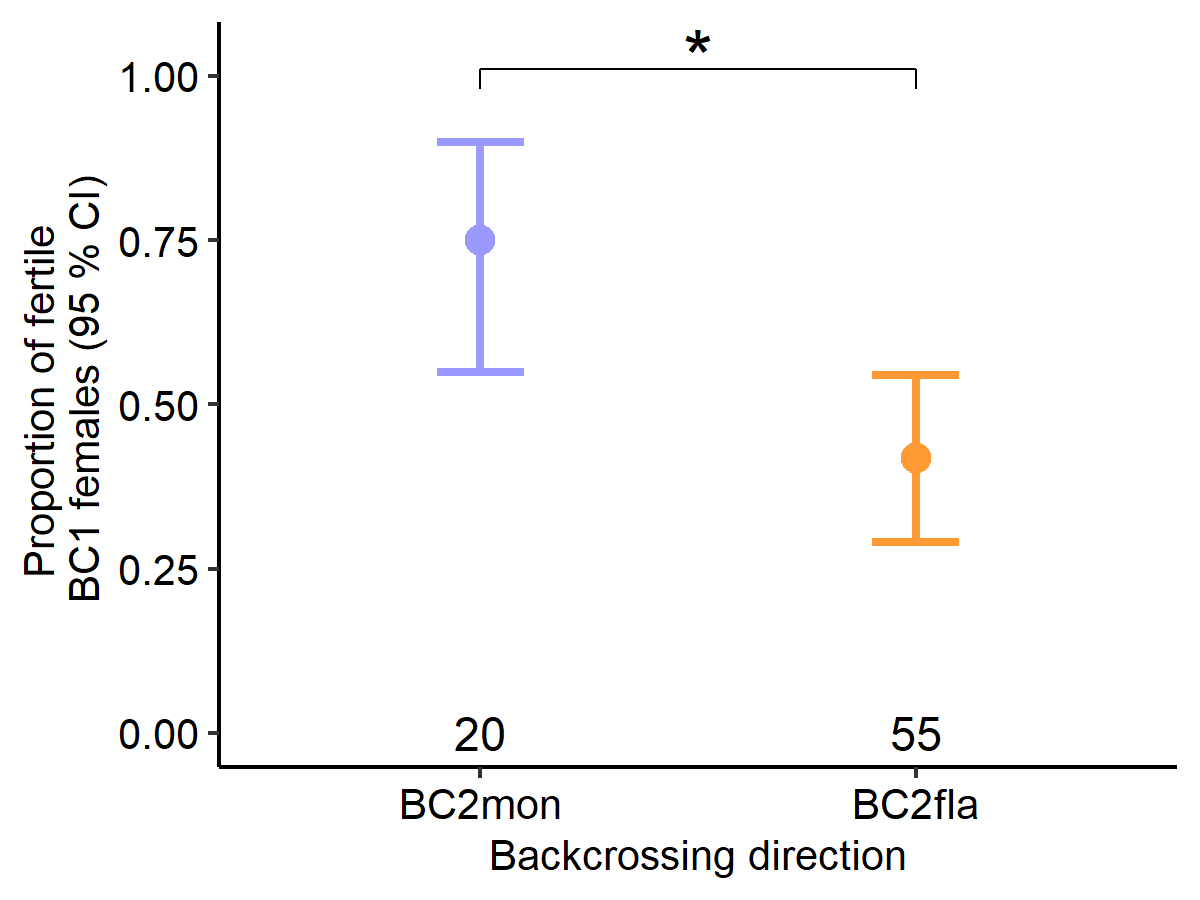


Figure S2. Proportion of fertile BC_1_ females from backcrosses between F_1_ females and *D. montana* males (blue) and between F_1_ females and *D. flavomontana* males (orange). Numbers below error bars refer to the number of tested BC_1_ females in each reciprocal cross. Error bars represent bootstrapped 95% confidence intervals (Mean ± CI).


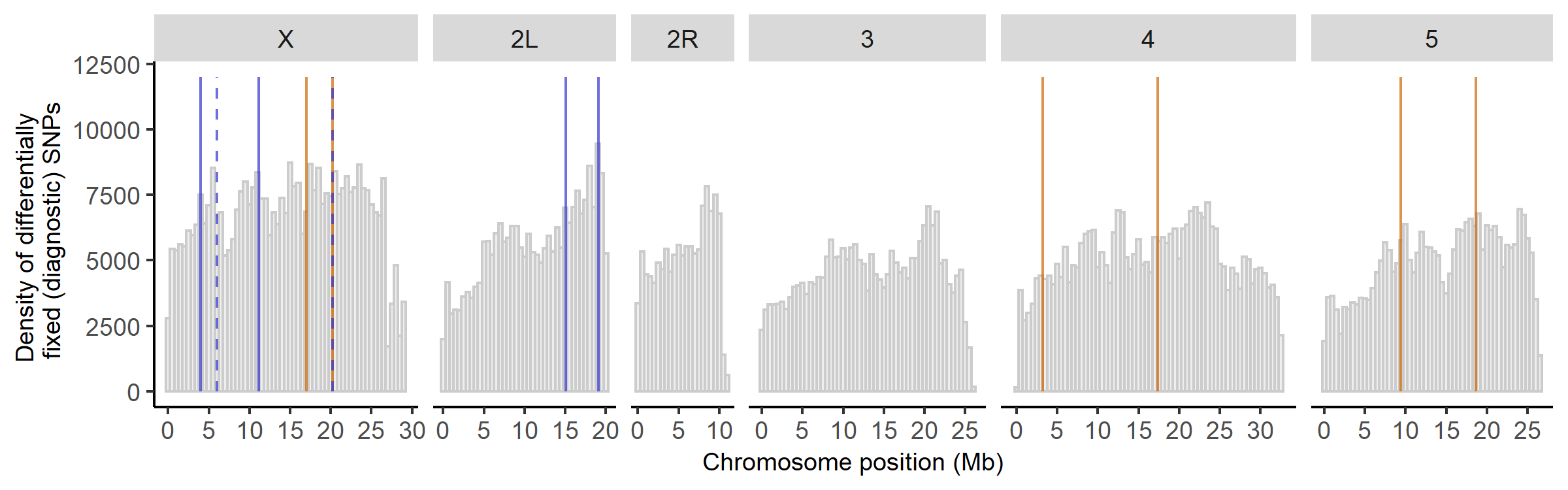
 Figure S3. Density of differentially fixed SNPs (in 200kb windows) between parental species across each chromosome (*D. montana* used as a reference genome). Orange and blue vertical lines represent species-specific *D. flavomontana* and *D. montana* chromosomal inversions, respectively. Solid and dashed vertical lines describe breakpoints of different inversions. Chromosome 2 involves left (2L) and right (2R) arms separated by a submetacentric centromere.


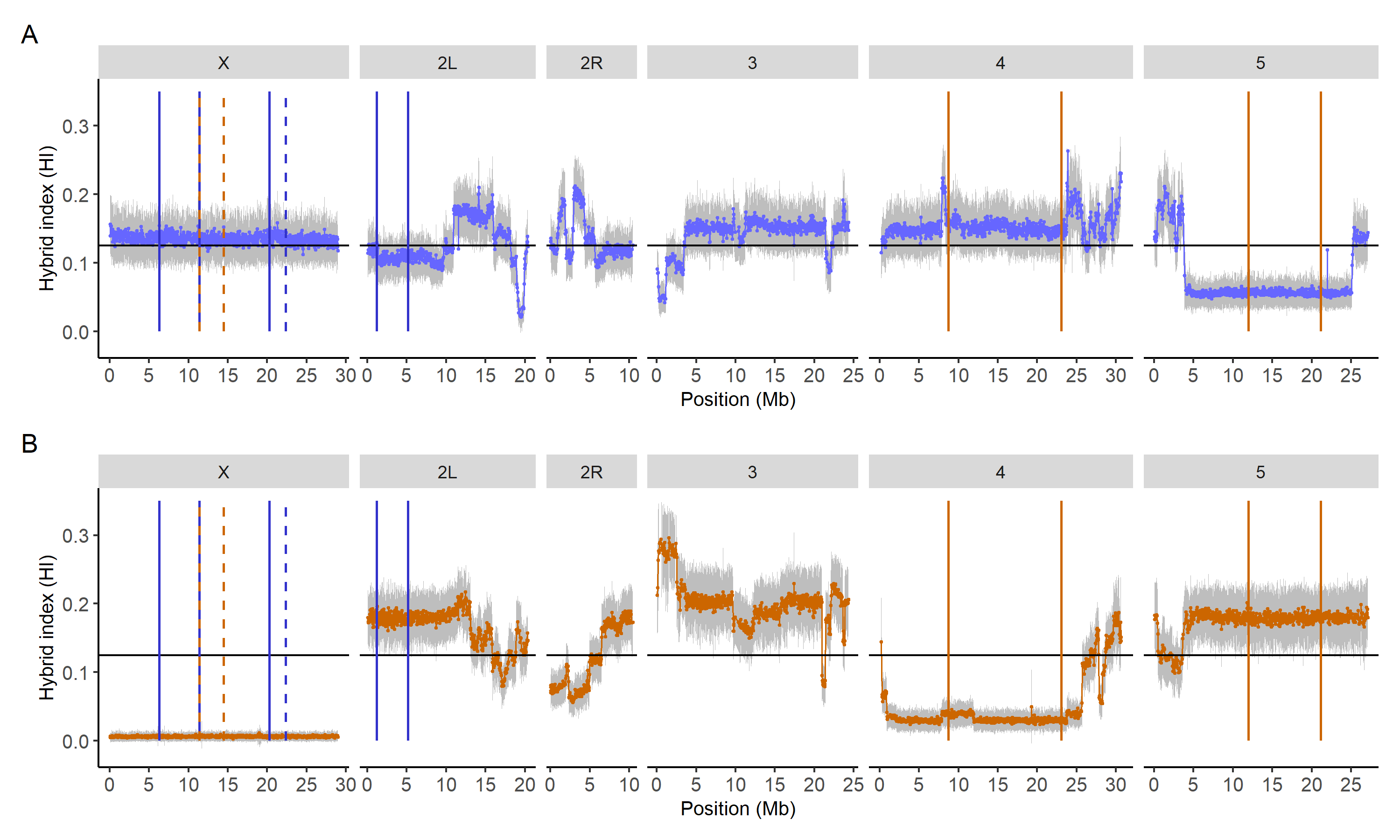
Figure S4. Observed hybrid index (HI) of 2^nd^ backcross generation female pools towards (A) *D. montana* (BC_2_mon) and (B) *D. flavomontana* (BC_2_fla). The data was averaged over windows of 400 non-overlapping SNPs along the genome. The grey area shows the variation within each averaged data point measured as standard deviation. The data is illustrated using the *D. flavomontana* reference genome. The left (2L) and right (2R) arms of chromosome 2 are separated by a metacentric centromere. The black horizontal line represents the expected amount of introgression, HI = 12.5 %, under neutrality. Vertical lines represent species-specific *D. flavomontana* (orange) and *D. montana* (blue) chromosomal inversions. Solid and dashed vertical lines show breakpoints of different inversions.


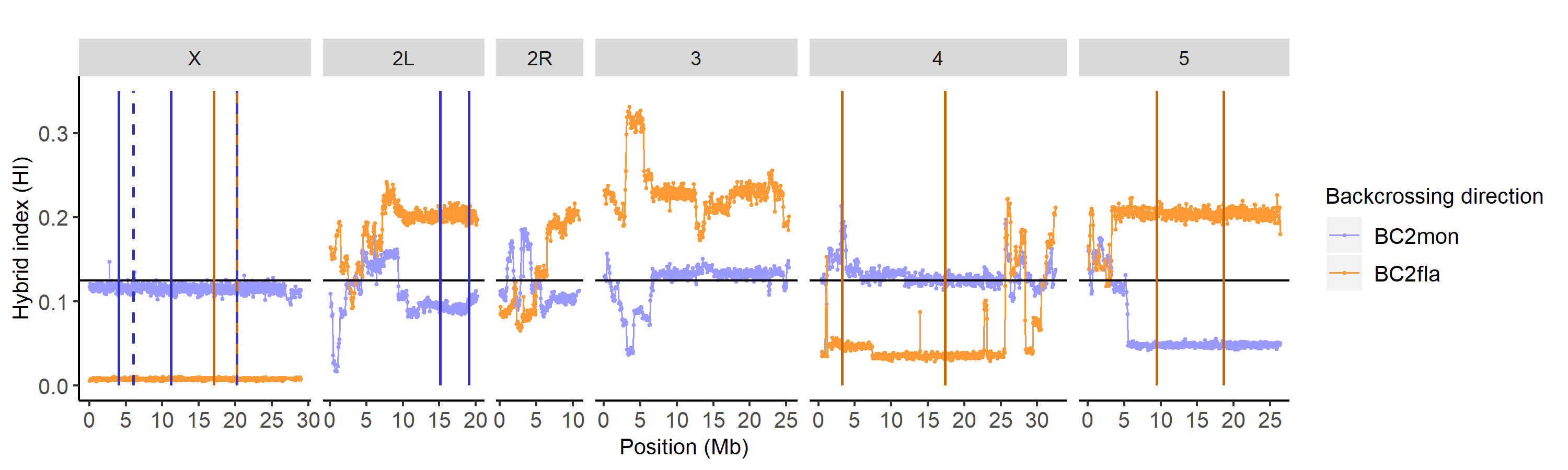
 Figure S5. Observed hybrid index (HI) of 2^nd^ backcross generation female pools towards *D. montana* (BC_2_mon) and *D. flavomontana* (BC_2_fla) in windows of 400 non-overlapping SNPs along the genome. The data is illustrated using the *D. montana* reference genome. For chromosome 2 the left (2L) and right (2R) arms are separated by a metacentric centromere. The black horizontal line represents the expected amount of introgression, HI = 12.5 %, under neutrality. Vertical lines represent species-specific *D. flavomontana* (orange) and *D. montana* (blue) chromosomal inversions. Solid and dashed vertical lines show breakpoints of different inversions.


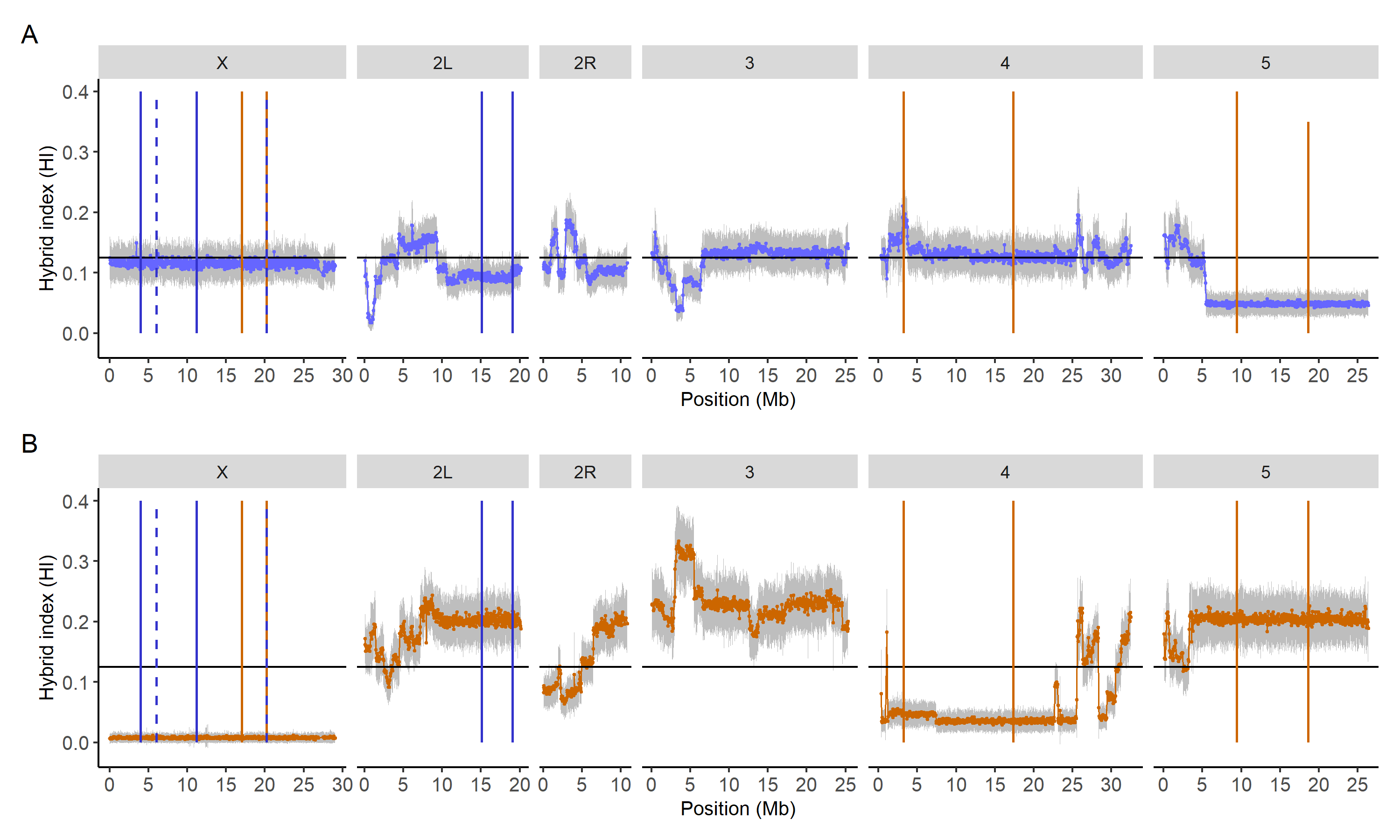
 Figure S6. Observed hybrid index (HI) of 2^nd^ backcross generation female pools towards (A) *D. montana* (BC_2_mon) and (B) *D. flavomontana* (BC_2_fla). The data was averaged over windows of 400 non-overlapping SNPs along the genome. The grey area shows the variation within each averaged data point measured as standard deviation. The data is illustrated using the *D. montana* reference genome. For chromosome 2 the left (2L) and right (2R) arms are separated by a metacentric centromere. The black horizontal line represents the expected amount of introgression, HI = 12.5 %, under neutrality. Vertical lines represent species-specific *D. flavomontana* (orange) and *D. montana* (blue) chromosomal inversions. Solid and dashed vertical lines show breakpoints of different inversions.


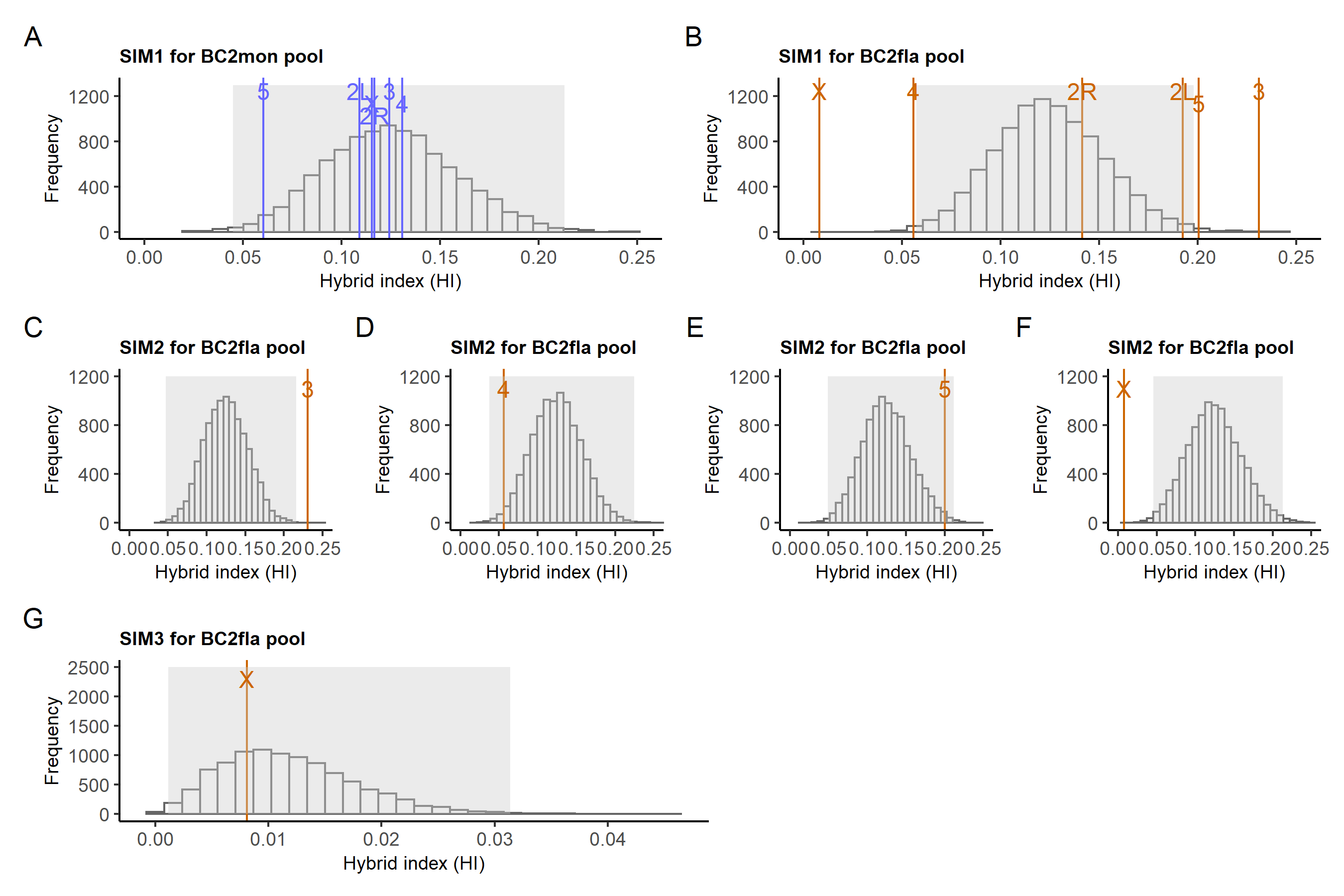


Figure S7. Hierarchical representation of the most meaningful simulations (10,000 replicates/simulation) of the 2nd generation backcross experiments towards *D. montana* (BC2mon) and *D. flavomontana* (BC2fla) (*D. montana* was used as a reference genome). The grey area of each figure represents Bonferroni corrected 5% and 95% quantiles and the space between them (regions beyond the area are statistically significant). Simulations under neutrality (SIM1) and the observed mean hybrid index (HI) of each chromosome for (A) BC_2_mon pool and (B) BC_2_fla pool. Simulations under neutral inversions (SIM2) and observed mean HI of BC_2_fla pool for (C) the 3^rd^ chromosome, (D) the 4^th^ chromosome, (E) the 5^th^ chromosome, and (F) the X chromosome. (G) Simulations involving inversions with a single locus against introgression (SIM3) and observed mean HI for the X chromosome of BC_2_fla pool.

Figure S8. Illustration of the expected allele frequencies of *D. montana* (A and B) after selecting a pairwise dominant-recessive incompatibility in hybrids backcrossed to *D. flavomontana*. A/a locus is dominant and B/b locus is recessive (note that we investigated only SNPs that were differentially fixed between the species). Red genotypes, involving dominant *D. montana* locus interacting with recessive *D. flavomontana* locus, are either sterile or inviable and will not contribute to the allele frequencies.

**Supporting References:**

Thorvaldsdóttir H, Robinson JT, Mesirov JP. 2012. Integrative Genomics Viewer (IGV): high-performance genomics data visualization and exploration. *Brief. Bioinform.* 14:178–192.
